# A GRU-Based Model for Detecting Common Accidents of Construction Workers

**DOI:** 10.3390/s24020672

**Published:** 2024-01-21

**Authors:** Ren-Jye Dzeng, Keisuke Watanabe, Hsien-Hui Hsueh, Chien-Kai Fu

**Affiliations:** 1 Department of Civil Engineering, National Yang Ming Chiao Tung University, Hsinchu 30010, Taiwan; as880916.en11@nycu.edu.tw; 2 Department of Marine Science and Ocean Engineering, School of Marine Science and Technology, Tokai University, Shizuoka 424–8610, Japan; keisuke@tsc.u-tokai.ac.jp

**Keywords:** sensor, accelerometer, fall detection, accident, construction worker

## Abstract

Fall accidents in the construction industry have been studied over several decades and identified as a common hazard and the leading cause of fatalities. Inertial sensors have recently been used to detect accidents of workers in construction sites, such as falls or trips. IMU-based systems for detecting fall-related accidents have been developed and have yielded satisfactory accuracy in laboratory settings. Nevertheless, the existing systems fail to uphold consistent accuracy and produce a significant number of false alarms when deployed in real-world settings, primarily due to the intricate nature of the working environments and the behaviors of the workers. In this research, the authors redesign the aforementioned laboratory experiment to target situations that are prone to false alarms based on the feedback obtained from workers in real construction sites. In addition, a new algorithm based on recurrent neural networks was developed to reduce the frequencies of various types of false alarms. The proposed model outperforms the existing benchmark model (i.e., hierarchical threshold model) with higher sensitivities and fewer false alarms in detecting stumble (100% sensitivity vs. 40%) and fall (95% sensitivity vs. 65%) events. However, the model did not outperform the hierarchical model in detecting coma events in terms of sensitivity (70% vs. 100%), but it did generate fewer false alarms (5 false alarms vs. 13).

## 1. Introduction

Fall accidents in the construction industry have been studied over several decades and have been identified as a common hazard and the leading cause of fatalities. In Taiwan, the Council of Labor Affairs reported that the construction industry accounted for 43–53% of all occupational accidental deaths, and fall accidents alone accounted for 23–33% of all accidental deaths [1]. The U.S. Bureau of Labor Statistics [2] reported that in 2021, nearly 1 in 5 workplace deaths occurred in the construction industry. Just over one-third of all construction deaths were caused by falls, slips, and trips. Of these, almost all were caused by falls to a lower level. The construction industry accounted for 46.2% of all fatal falls, slips, and trips in 2021.

As sensor hardware and artificial intelligence rapidly advance today, sensors attachable to the human body have been used to capture human motion. Motion-capture technologies typically recognize human actions by capturing the sensed action data of a target because computer systems and humans do not understand raw data without further analysis. The technologies used to recognize the actions of construction workers can be classified into vision- and non-vision-based technologies. Vision-based technologies convert raw video images of an observed target person into computerized data that can be understood by the designed system. They include marker-based (e.g., optical system) and marker-less (e.g., RGB-depth cameras) technologies. In general, compared to marker-less systems, marker-based motion-capture systems are more expensive, require complex setups, and interfere more with workers’ activities. However, they offer higher accuracy than marker-less systems and avoid occlusion problems in some cases.

Non-vision-based technologies identify human actions without visual perception of a target person, and they usually use inertial sensors, such as accelerometers, gyroscopes, and pressure sensors, to sense the actions of the target person. Accelerometers, occasionally coupled with gyroscopes, are widely used to track human actions in the construction industry because they are mobile, wearable, and suitable for the complex working environments of construction sites. In addition, they eliminate the problems of visual obscuration.

Meanwhile, inertial measurement units (IMU) have been used in different application domains, such as physical activity monitoring for individual fitness [3] or sports performance [4]. Jones et al. [5] used the accelerometer data of 85,670 participants from the UK Biobank and performed a genome-wide association study of eight derived sleep traits representing sleep quality, quantity, and timing. Similarly, accelerometers were also used to analyze human gait patterns for detecting walking abnormalities to help assess musculoskeletal conditions [6] and improve rehabilitation progress [7]. Arias et al. [8] characterized the number of minutes of moderate and vigorous physical activity at work and outside of work during 7 consecutive days by studying the data of 55 commercial construction workers.

In addition, IMUs have been used to recognize various activities at construction sites. For example, Sanhudo et al. [9] used wearable accelerometers and supervised machine learning algorithms to classify 10 different activities (e.g., gearing up, hammering, masonry, painting, sawing, screwing, and sitting) in a simulated laboratory environment. A few researchers have focused on detecting awkward postures that contribute to work-related musculoskeletal disorders. For example, Nath et al. [10] used built-in smartphone sensors to unobtrusively monitor workers’ bodily postures and autonomously identify potential work-related ergonomic risks. Arias et al. [8] used accelerometers to monitor construction workers’ activities, but they focused on classifying moderate and vigorous physical activity at work and outside of work.

Finally, IMUs have been used to detect falls, trip accidents, or other portents that possibly contribute to accidents at construction sites. For example, Dzeng et al. [11] were the first to study the feasibility of using multiple accelerometers and gyroscopes to detect falls and fall portents associated with tiling activities without unnecessary movements in limited scaffold spaces. Fang and Dzeng [12] continued development work on a smartphone-based personal safety monitoring system. This system received external signals wirelessly from motion sensors attached to a vest at the chest position, waist, and arm, as well as a set of brain wave sensors inside a helmet, and transmitted these signals to a monitoring server for further analysis. They proposed an algorithm and experimented to detect falls, trips, and portents (e.g., heavy footsteps, sudden knee movements, sudden swaying, abrupt body reflexes) by considering four different physiological statuses of the subjects (i.e., sleepiness, fatigue, normal, and inebriation). Achour et al. [13] developed an accelerometer-data-based algorithm to detect worker falls with a focus on reducing power consumption by using sensor timers.

In summary, several IMU-based systems for detecting fall-related accidents have been developed and have yielded satisfactory accuracy. However, in almost all of the related studies, experiments were conducted in well-controlled simulated laboratory environments, and these experiments involved limited worker activities. Unlike production factories, the working environments of construction workers are far more complex with unpaved, uneven grounds and many temporary or unfinished structures (e.g., scaffolding, rough floor). These noises may affect the detection performance of IMU-based systems tremendously. Similar to other researchers, the corresponding author’s team developed an IMU-based system to detect falls and related portents for construction workers and achieved satisfactory performance in a simulated laboratory environment. However, when the system was applied to real construction projects, it produced many false alarms, which made it impractical for use in such projects.

In the present work, first, two versions of previously developed algorithms for detecting falls and stumbles of construction workers are reviewed. Then, the findings obtained by applying one of the existing systems to a building and three real riverbank conservation projects are presented. Finally, the paper describes how the authors solve the problem of false alarm generation that occurs when the system is applied to real projects by redesigning the experiment and redeveloping the system based on the gated recurrent unit (GRU) deep machine learning model.

## 2. Review of Previous Developments

In this section, the two systems for detecting falls and related portents that were previously developed by the authors are summarized.

### 2.1. SVM-Threshold-Based System (SVM-S)

**Figure 1:**
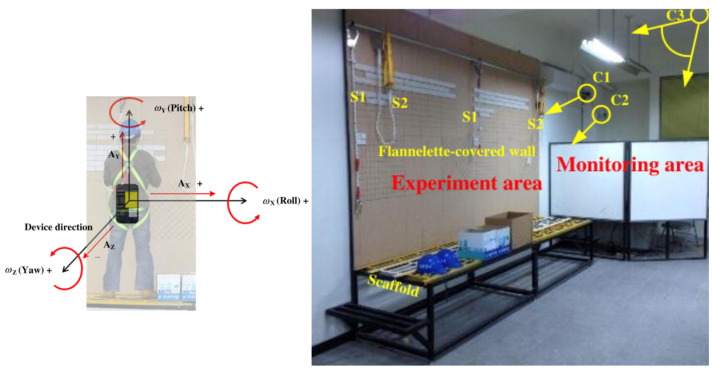
Experimental setting for SVM-S.

This system uses data from a single accelerometer and gyroscope embedded in a smartphone attached to the back of a worker’s vest (close to the waist area), as shown in the left of Figure 1, to detect falls and the related portents. An experiment was conducted in which a tiling operation was performed in a simulated scaffolding inside a laboratory, as shown in the right of Figure 1.

Algorithms based on the threshold of signal vector magnitude (SVM) were used to detect falls and their portents, including the SVMs of an accelerometer and a gyroscope, as well as the difference between the SVMs of the accelerometer at consecutive time points. The experiment concluded that the SVM of the accelerometer and the difference between the SVMs of the accelerometer at different time points exhibited the best specificity (99.9% and 99.7%, respectively), and both exhibited 100% sensitivity. The SVM of the gyroscope led to the worst performance. Additionally, the authors pointed out that the high specificity and sensitivity were not good indicators to determine whether the system was suitable for field application because the number of unsafe events (i.e., alarm targets) were submerged by the comparatively very large number of safe events (i.e., normal events). Therefore, they proposed an accuracy rate (i.e., number of correct detections divided by the number of alarms generated) and a false detection rate (i.e., number of incorrect detections divided by the number of alarms generated) to avoid the effect caused by the large number of normal events. As a result, the experiment yielded an accuracy rate of 88.5% and a false detection rate of 11.5%, and the authors concluded that the use of accelerometers to detect fall-related accidents of construction workers was feasible. For more details, please refer to [11].

### 2.2. HiERarchical threshold System (HER-S)

Fang et al. [12] proposed an advanced fall portent detection system that combined multiple accelerometers with hierarchical thresholds. The experimental setup was as follows: the participants wore accelerometers on their arms, wrists, chests, and waists. The system integrated commonly used SVM and vertical acceleration algorithms to identify fall-related portents, including heavy footsteps, sudden knee movements, sudden swaying, abrupt body reflexes, imbalance, saving tile drops, and unsteady footsteps. The detection of these portents was more difficult than fall detection. The experiment was conducted in a similar simulated environment in a laboratory, with the participants freely performing the specified tile-laying task under different conditions, including normal daytime and nighttime conditions, as well as under the influence of alcohol. It was concluded that the waist position was the best sensor position, and the optimal algorithm yielded sensitivity and specificity values of 95.2–98% and 100%, respectively, for detecting direct falls. In addition, its accuracy and detection rate for fall-related portents were 79.13% and 76.86%, respectively.

### 2.3. Lessons Learned from Actual Field Use

The authors had the opportunity to apply HER-S to real projects, including three rive-bank conservation projects with 25 participating construction workers, and one building project with 10 participating construction workers. However, the feedback obtained from the actual projects was not as positive as the experimental results owing to unexpected complex environments and noise situations in the projects.

During the application period, no accidents, including *stumbles*, *falls*, and *coma*, occurred. However, many false alarms were generated. Table 1 lists the major false alarms generated during the field use of HER-S and the reasons for their generation, which were suggested based on observations. Problems 1–5 are false-positive problems, and problems 6–7 are false-negative problems. However, the so-called “positive” cases in the field were simulated cases because no accident occurred during the test period. As explained in the table, Problems 5 and 7 resulted from incorrect usage (e.g., workers should disable the alarm app when resting or leaving the smartphone on a table), and they can be solved by asking and reminding the workers to abide by the rules of usage. Therefore, in this work, the authors aim to address the false alarms reported in the table, except Problems 5 and 7.

## 3. Methodologies

Thanks to the advancement of graphical processing units in recent years, it has become possible to use deep neural networks for solving complex classification problems in diverse domains. Among them, convolution neural networks (CNN) and recurrent neural networks (RNN) are two of the most commonly used models. While CNN has been mainly used for image recognition, RNN is suitable for processing time- or space-dependent data series, such as natural language processing. In this study, RNN is used to classify the time series of IMU data for identifying alarm events such as *stumble*, *fall*, and *coma*.

Despite the widespread use and promising performance of RNN, the conventional RNN cannot capture long-term memory, and it is affected by the vanishing gradient problem during the process of propagation through various network layers. Hochreiter and Schmidhuber [14] proposed long short-term memory (LSTM) to better retain important long-term memories during the propagation process. In this study, intensive sampling of IMU readings led to the accumulation of a large amount of data. For instance, an event with a 2 s window may generate 20 time units of data series, and retaining these data at the initial time unit could be essential for target classification. Nevertheless, while LSTM can retain long memory, the initial data trial in this study was time consuming owing to the large volume of data generated by high-frequency sampling. Chung et al. [15] proposed the gated RNN by using the GRU to improve propagation efficiency and reduce memory loading while retaining long-term memory. Therefore, in the present study, the GRU version of LSTM is employed.

### 3.1. Problem Statement

Given the accelerometer, gyroscope, and magnetometer data generated by the built-in IMUs of the smartphones worn by the participating workers, the system generates alarms when the workers *stumble*, *fall*, or are in a *coma*; alternatively, it shows the *safe* status when an accident does not occur. The objective of the present research is to reduce false alarms while maintaining the same detection accuracy as that of HER-S.

### 3.2. Research Design

**Figure 2:**
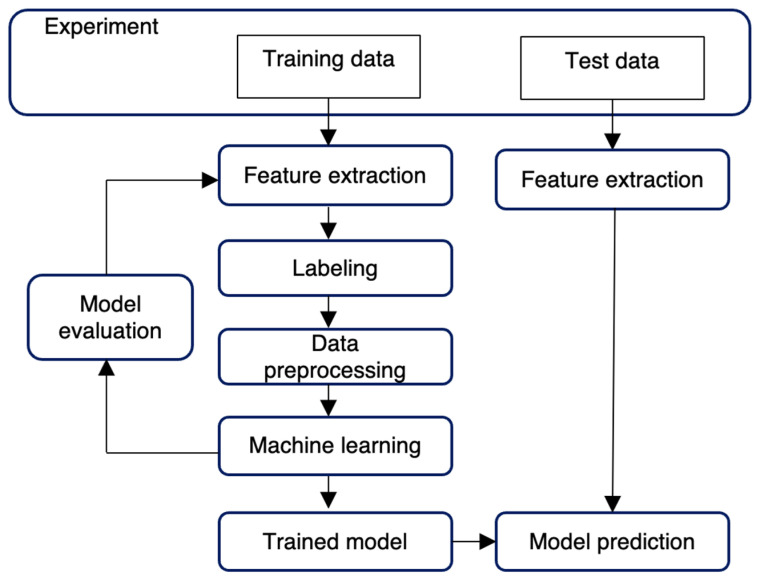
Research design.

Figure 2 depicts the research process. First, an experiment simulating the construction site environment outside the laboratory is designed, and two sets of IMU data for training and testing purposes are collected experimentally. Then, features are extracted from the training data. These extracted features are labeled with correct answers and fed to the GRU model. After training is complete and the validation criteria are fulfilled, the trained model is considered ready for classifying workers’ movement types. The test data are used to test and evaluate the performance of the model.

### 3.3. Experiment Design

The objective of this study is to improve the detection accuracy of the system when it is used in real projects. Because it is unsafe and impractical to collect data and observe workers’ unsafe events in actual construction sites without interrupting their work, the project selected for the experiment must at least simulate the environment in which the reported types of false alarms (Table 1) are triggered easily. The authors identified several types of environments on their university campus, including uneven roads, inclined and abrupt slopes, stairs, and straight and curved motorcycle lanes with speed bumpers. The participants were asked to perform the following tasks under surveillance.

1. Walking on even and uneven roads, climbing four flights of stairs, and walking on inclined slopes in and around the department building.
2. Walking on an uneven gravel trail and on inclined and abrupt slopes for 1.5 min in the forest located on the campus.
3. Riding a motorcycle in the motorcycle lanes on the campus, which include straight and curved lanes with bumpers.
4. Simulating falls by raising a plush doll to which a 1 kg weight and a smartphone are attached, allowing the doll to sway a few times, and then dropping it from the half-story height of a bookshelf, as well as from the one-story height of a stairway.
5. Simulating a stumble by deliberately falling on an upholstered floor surface in the forward, left-side, right-side, and rear directions.
6. Simulating progressive coma by resting, sitting down, and going into a simulated coma (i.e., remaining still) for at least 30 s. Simulating abrupt coma by going into coma directly from a standing position.
7. Resting freely in a sitting position for 2 min.

### 3.4. Data Preprocessing

Thirty students participated in the experiment. The data series collected from each participant included seven pieces of information: date, time, accelerometer data along the X, Y, and Z axes, pitch angle, and roll angle. These data were additionally labeled with the type of *status* (i.e., 0, 1, 2, and 3 for *safe*, *fall*, *stumble*, and *coma*, respectively) based on post-observation of recorded surveillance videos. Useful annotations such as “impact with the ground after fall” that may be helpful for post analysis were attached to the corresponding time frames. These annotations were used only for human analysis, not for machine learning.

The initial amount of data collected was huge and required reduction. The sampling frequency of the sensors was set to 0.1 s. Consequently, each participant generated approximately 5500 data series in the entire experiment. With a total of 30 participants, this resulted in 165,000 data series. Furthermore, each data series included seven pieces of information (e.g., date, time, accelerometer data). This led to a total dataset size of 1,155,000 data points.

Considering the duration of the target events (e.g., *fall*, *stumble*), the authors arbitrarily set 2 s (i.e., 20 data series with a 0.1 s sampling rate) as the data sliding window with a stride of 1 for determining the type of an event and the label of the 10th data series as the event’s status. For example, the first event is composed of the 1st to the 20th data series, and the status label of the 10th sequence is the *status* of this event. The second event is composed of the 2nd to the 21st data series, and the label of the 11th is its status.

### 3.5. Machine Learning Model

The GRU machine learning model was implemented on the Kaggle platform [16] by using Python programming language. The model mainly used the *sequential* and *keras.layes* modules of the *keras.models* package. The GRU built herein was composed of five hidden layers (i.e., *dense* layers), two *dropout* layers, and one *output* layer.

The activation function of the model’s hidden layer was rectified linear unit (*ReLU*), which is commonly used as an activation function for hidden layers, along with others such as *sigmoid*, *tanh*, and *Leaky ReLU*. The *sigmoid* function is susceptible to the vanishing gradient problem and outputs non-zero-centered output values, which causes all weights to become either positive or negative when all inputs are positive. *Tanh* improves upon the non-zero centering issue of *sigmoid*, but it does not fully address the vanishing gradient problem. Glorot et al. [17] proposed *ReLU*, which solves the vanishing gradient problem. However, *ReLU* is susceptible to neuron death when the input values are negative. Therefore, *Leaky ReLU* was developed to address this problem. *ReLU* was selected in this study because all the inputs were positive values. In what follows, the GRU-based system developed herein is called GRU-S.

## 4. Results

### Experiment Result

**Figure 3:**
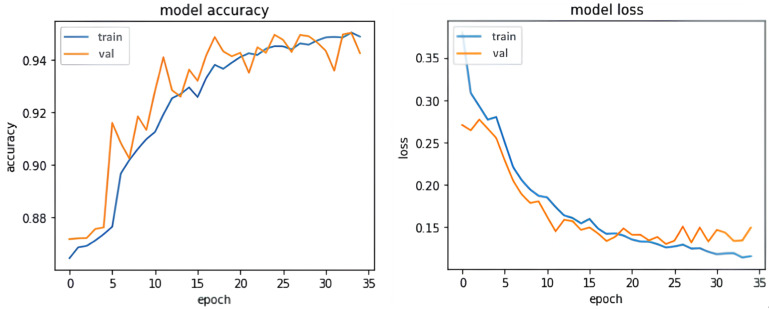
Accuracy and loss of GRU-S during training and validation.

Among the 30 participants, the data of 25 randomly selected participants were used to train the model, and the rest of the data were used to test the trained model. Figure 3 shows the accuracy and loss of the model during the training and validation phases.

**Figure 4:**
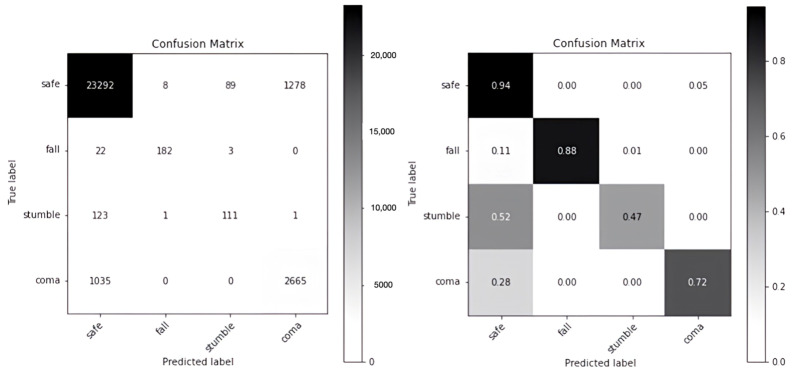
Confusion matrix of GRU-S.

**Figure 5:**
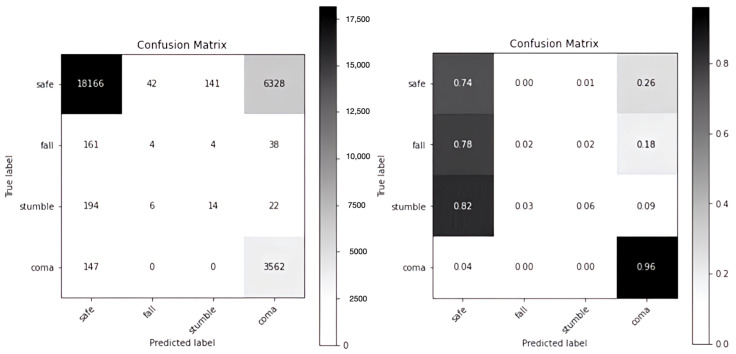
Confusion matrix of HER-S.

Figure 4 shows the results of the model in terms of time-unit data in the form of the confusion matrix of GRU-S as numbers (left) and the corresponding percentages (right). For example, for the *safe* label, the accuracy percentage is 0.94 (=23292/(23292 + 8 + 8 + 89 + 1278)). For the target alarm labels of *fall*, *stumble*, and *coma*, the accuracies were 0.88, 0.47, and 0.72, respectively. Figure 5 shows the confusion matrix of the previously developed HER-S for the same set of data. For the target alarm labels of *fall*, *stumble*, and *coma*, the accuracies are 0.02, 0.06, and 0.96, respectively. Apparently, GRU-S performed better than HER-S in accurately detecting the *fall* and *stumble*, but its performance was poorer for *coma*.

Because the participants were in a *safe* condition for most of the time, an unbalanced dataset was collected, that is, the number of target events was considerably smaller than that of non-target events (i.e., *safe*). Therefore, the high detection accuracy of the *safe* label (0.94) did not mean much in practice. We further used the F1 measure to compare the two models.

Table 2 and Table 3 list the F1 scores and other accuracy measures of both systems. *Accuracy* is the most commonly used metric, and it indicates the proportion of correct predictions made by a model (excluding both type I and type II errors). It is calculated as TP + TN/(TP + TN + FP + FN) (TP = true positive, TN = true negative, FP = false positive, and FN = false negative). While both *precision* and *recall* focus on TP, they offer different perspectives. *Precision* measures the accuracy of a model in correctly identifying instances of a particular action taken by the subject: TP/(TP + FP). Meanwhile, *recall* measures the proportion of actual instances of a particular action taken by the subject that are correctly identified by the system: TP/(TP + FN). *Specificity* focuses on TNs, and it measures the accuracy of the system in not identifying a particular action when a subject did not perform it: TN/(TN + FP). F1 Score is the harmonic mean of *precision* and *recall*, and it provides a balanced view of the performance of these two metrics simultaneously.

In Table 2, GRU-S performed well on all or most of the *accuracy*, *precision*, *recall*, and *specificity* measures in cases of *safe*, *fall*, *stumble*, and *coma* events. However, on the *precision* and *recall* indexes, the model did not perform well in cases of the *stumble* and *coma* events. Despite this relatively poor performance, GRU-S outperformed HER-S (Table 3) in detecting *stumble* and *coma* on the *precision* index (i.e., 54.68% vs. 8.81% and 67.57% vs. 35.80%) and in detecting *stumble* on the *recall* index (47.03% vs. 4.93%). In terms of the F1 Score, GRU-S outperformed HER-S.

Since the purpose of this study is to reduce the number of false alarms, Table 4 summarizes the performances of both GRU-S and HER-S in terms of accurate detection of target events and false alarms during *safe* events. GRU-S outperformed HER-S in detecting *falls*. GRU-S successfully detected all *falls* (100% sensitivity), triggering only three false alarms. By contrast, HER-S detected only 4 out of 10 falls while mistaking three *falls* as *stumbles*. Moreover, it missed 3 *falls* and triggered 85 false alarms. For *stumble*, GRU-S outperformed HER-S. GRU-S detected 19 out of 20 *stumbles* (95% sensitivity) and triggered 35 false alarms. By contrast, HER-S detected only 13 *stumbles* (65% sensitivity) and triggered 124 false alarms.

Nevertheless, surprisingly, GRU-S underperformed HER-S in detecting *coma*. GRU-S detected 7 out of 10 *comas* (70% sensitivity) and triggered 5 false alarms. HER-S detected all *comas* (100% sensitivity) but triggered 13 false alarms, which was far greater than that triggered by GRU-S.

## 5. Discussion

In general, GRU-S outperformed HER-S, except in detecting *comas*. A post-review of the recorded videos was performed to identify the situations in which GRU-S tended to make mistakes. *Stumbles* with obvious kneeling first (i.e., a two-step process instead of continuous stumbling and falling) tended to confuse GRU-S more than other types of *stumbles*. Among the 124 and 85 false alarms related to *stumble* and *fall*, the situations that triggered the highest numbers of alarms were quick or sudden *safe* movement with abrupt accelerometer movements, such as walking on stairs or uneven surfaces and riding a motorcycle over potholes. Among the 35 and 3 false alarms related to *stumble* and *fall*, the situations that triggered the highest numbers of alarms were unsteady walking or abrupt heavy stepping or stomping when walking on uneven or sloping surfaces.

Although GRU-S underperformed HER-S in detecting *comas*, it triggered fewer false alarms (5 vs. 13). GRU-S produced FPs of *coma* only when the subjects were resting, while HER-S tended to trigger FPs when the subjects were resting or stopping when riding a motorcycle.

In what follows, the data of the three main types of false alarms are analyzed further, and possible remedial strategies for future improvement of GRU-S are proposed.

### 5.1. False Identification between Fall and Stumble

**Figure 6:**
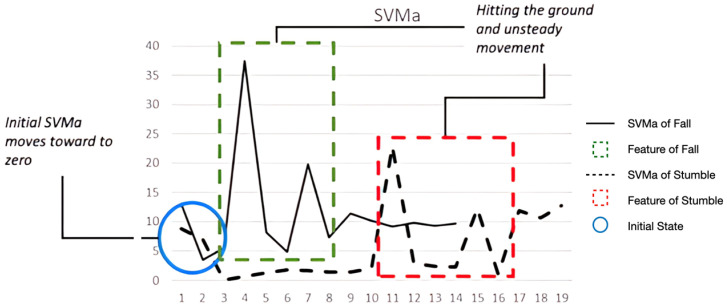
Comparison of *SVMa* between a *fall* and a *stumble*.

Figure 6 shows examples of *SVMa* data (i.e., SVM of accelerometer data) for the *fall* and *stumble* events. Although the two polylines have different magnitudes and time spans, they resemble each other in terms of patterns, which may lead to misidentification. Both *fall* from height and *stumble* are initiated with a fall, resulting in *SVMa* values closer to zero first. Then, when the subjects hit the ground either because of a *fall* from height or a *stumble*, *SVMa* increases abruptly and reaches its peak owing to the counterforce produced by the ground and, finally, follows the patterns corresponding to unsteady movements.

Such misidentifications accounted for less than 5% of the detection results and did not seem to cause problems in the experiment. However, to improve the accuracy of distinguishing a *fall* from a *stumble*, more realistic data must be collected, if possible, and the *SVM* magnitudes of the two situations must be studied. One could also study the *SVMa* patterns of the following actions after the events. The *fall* of interest of this study is fall from height, which usually causes severe harm that immobilizes a person at least temporarily. Thus, standing and walking events are unlikely to be detected in the following actions. Conversely, larger movements, standing, or walking patterns are likely to be detected following a *stumble*. Therefore, observing the difference between the actions after *fall* and *stumble* will help to distinguish between them.

### 5.2. False Identification between Safe and Stumble

GRU-S mis-detected a few *safe* situations as *stumbles*, but this did not significantly affect its overall detection accuracy. These mis-detections usually occurred when the subjects walked on uneven sloped surfaces. The authors believe that accurately distinguishing such safe walking situations from the stumble situations based on IMUs alone is difficult because the corresponding inertial data patterns are similar, and the differences between their magnitudes are ambiguous. The study and detection of the differences between the possible actions that follow the two situations might be more practical than detecting the two situations directly. For example, walking on uneven surfaces usually results in the same false *stumbles* for a longer time, while the *stumble* pattern of a true *stumble* lasts only 2–3 s.

### 5.3. False Identification between Coma and Rest

This type of misidentification was the main problem of GRU-S. Since the *coma* data were collected by the subjects simulating the *coma* conditions, the authors argue that the system’s underperformance cannot be concluded decisively, and the system could perform better when it encounters a true coma. Because the subjects were not really in a coma, they might have made slight movements invisible to the naked eye but were detectable by IMUs. Therefore, the three misidentifications of coma by GRU-S (Table 4) could be TNs because the subjects were unable to maintain complete stillness.

**Figure 7:**
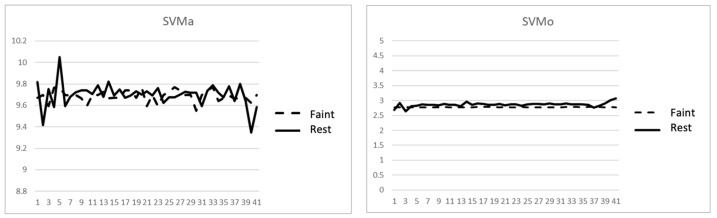
Comparison of SVMa and SVMo between *coma* and rest.

Suppose the subjects did maintain complete stillness during the experiments (i.e., all 10 *comas* were true *comas*), and GRU-S did underperform HER-S. The situations that caused GRU-S to fail in identifying *comas* were those in which the subjects pretended to faint by gradually sitting down first before entering the coma. Figure 7 shows comparisons of SVMa and SVMo (SVM of rotation angle) between the fainted-while-sitting and sitting-and-resting poses of a subject over a 4 s timeframe. The resemblance between the two situations makes it difficult to distinguish between them based on inertial data alone. However, the two situations did not resemble each other over longer periods (e.g., 30 s) because the subjects tended to move slightly when resting unless they were asleep. Thus, one way to distinguish the fainted-while-sitting situation from the sitting-and-resting situation is by providing the model with a higher-level decision-making mechanism based on the observation of a longer timeframe. For example, the current system generates a detection label every 2 s. Monitoring a human who has really fainted while sitting for a minute would more likely result in a steady series of 30 *coma* labels. Meanwhile, monitoring a sitting and resting human would more likely result in a few *safe* labels among *coma* labels because of slightly unavoidable movements. The difference between the two patterns over a longer timeframe could increase the feasibility of distinguishing between the corresponding situations.

Another approach is to integrate GRU-S and HER-S, make GRU-S responsible for detecting only *stumble* and *fall*, and make HER-S responsible for detecting only *coma*. However, by replacing GRU-S with HER-S for detecting *coma,* a greater number of false alarms will be generated than when using GRU-S alone. As a solution, either this tradeoff must be accommodated or the threshold of HER-S must be studied further and adjusted.

## 6. Conclusions

This paper reviewed existing applications of IMUs to detect common accidents of workers at construction sites (i.e., *stumble*, *fall*, and *coma*). The various algorithms reviewed herein performed well in simulated working environments inside laboratories. However, they generated too many false alarms in practical environments because of the complexity of these environments and workers’ behaviors (e.g., walking on uneven sloped surfaces or stairways, or riding motorcycles on site). In this research, an existing algorithm (i.e., hierarchical threshold algorithm) was applied to real construction sites, and the situations that tended to trigger false alarms were identified. Based on the feedback obtained from these environments, the authors redesigned and conducted the aforementioned simulated experiment outside the laboratory, targeting situations prone to false alarms. A new GRU-based system, GRU-S, was developed to reduce the frequencies of various types of false alarms in outdoor environments.

GRU-S outperformed the existing benchmark model, HER-S, with higher sensitivities and fewer false alarms in detecting *stumble* (100% sensitivity vs. 40%, 3 false alarms vs. 85) and *fall* (95% sensitivity vs. 65%, 13 false alarms vs. 124). However, it fared poorer than HER-S in detecting *coma* in terms of sensitivity, but it triggered fewer false alarms (70% sensitivity vs. 100%, 5 false alarms vs. 13). Assuming there were no slight movements that were invisible to the naked eye when the subjects enacted the *coma* status, and GRU-S did underperform HER-S, the discussion section outlines two possible approaches for future research to solve this problem, namely deployment of higher-level decision-making mechanisms with longer-timeframe observations and integration of GRU-S with HER-S.

## Figures and Tables

**Table 1:** Main problems reported from field usage of HER-S.

No.	Problem Reported	Reasons
1	The *stumble* alarm was triggered sometimes as workers walked on site.	The construction sites, especially those of the riverbank projects, with uneven surfaces and gravel tended to cause unbalanced body movements during walking.
2	The *stumble* alarm was triggered sometimes when climbing down stairs.	Owing to step height difference, the movement of climbing down stairs tended to generate accelerometer signals similar to that of falling.
3	The *stumble* or *fall* alarm was triggered sometimes when riding a motorcycle.	Riding a motorcycle on a rough road or ground tended to generate large-magnitude accelerometer signals that were easily detected as *stumble* or *fall*.
4	The *coma* alarm was triggered sometimes when resting.	Resting led to the generation of extremely small-magnitude signals over long periods, which were similar to the signals generated when a person was in a *coma*.
5	The *coma* alarm was triggered sometimes when in a meeting.	The designated smartphones were attached to the back side of workers’ safety vests to minimize the inconvenience possibly caused to workers when performing their jobs. When in a meeting, some workers took off their vests and placed them on a table, resulting in generation of the *coma* alarm. The workers were supposed to disable the alarm app when not working.
6	Some simulated stumbles triggered *fall* alarms.	When the triggered threshold was set to a high value, a few *stumbles* would not be detected. However, when the threshold was set to a low value, false *stumble* alarms might be generated.
7	Some simulated falls from a height of 45 cm did not trigger the *fall* alarm or trigger the *stumble* alarm instead.	In practice, falls from a height of only 45 cm were not the events that the research attempted to identify. However, for safety purposes, only the data of simulated falls from a height of 45 cm were available. The number of false alarms of this type should be reduced greatly for true hazardous falls, which usually occur from a height of at least one story.

**Table 2:** F1 Score of GRU-S.

Label	Accuracy	Precision	Recall	Specificity	F1 Score
*safe*	91.13%	95.18%	94.43%	71.52%	0.948
*fall*	99.88%	95.29%	87.92%	99.97%	0.915
*stumble*	99.25%	54.68%	47.03%	99.68%	0.506
*coma*	91.97%	67.57%	72.03%	94.91%	0.697

**Table 3:** F1 Score of HER-S.

Label	Accuracy	Precision	Recall	Specificity	F1 Score
*safe*	75.67%	97.31%	73.62%	87.91%	0.838
*fall*	99.13%	7.69%	1.93%	99.83%	0.031
*stumble*	98.73%	8.81%	5.93%	99.49%	0.071
*coma*	77.33%	35.80%	96.04%	74.57%	0.522

**Table 4:** Accurate detection rate and number of false alarms for target events.

Model	Performance Index	*Fall*	*Stumble*	*Comma*
number of target events		10	20	10
GRU	number of accurate detection	10	19	7
	Sensitivity (%)	100%	95%	70%
	number of false alarms	3	35	5
Hierarchical	number of accurate detection	4	13	10
	Sensitivity (%)	40%	65%	100%
	number of false alarms	85	124	13

## Data Availability

The datasets presented in this article are not available because they have been destroyed according to the informed consent of IRB for this research.
